# CT Radiomics-based machine learning approach for the invasiveness of pulmonary ground-glass nodules prediction

**DOI:** 10.1016/j.ejro.2025.100680

**Published:** 2025-08-23

**Authors:** Rui Chen, Hu Zhang, Xingwen Huang, Haitao Han, Jinbo Jian

**Affiliations:** a Department of Oncology, Binzhou Medical University Hospital, Binzhou, Shandong 256603, PR China; b Department of Radiology, Binzhou Medical University Hospital, Binzhou, Shandong 256603, PR China; c Department of Cardiovasular Medicine, Binzhou Medical University Hospital, Binzhou, Shandong 256603, PR China; d Department of Radiology, Yantai Yantaishan Hospital, Yantai, Shandong, 264000, PR China

**Keywords:** Pulmonary ground-glass nodules, Lung adenocarcinoma, Pathological subtyping, CT radiomics, Machine learning

## Abstract

**Objective:**

To develop and validate a machine learning model based on CT radiomics to improve the ability to differentiate pathological subtypes of pulmonary ground-glass nodules (GGN).

**Methods:**

A retrospective analysis was conducted on clinical data and radiological images from 392 patients with lung adenocarcinoma at Binzhou Medical University Hospital between January 1, 2020 to May 31, 2023. All patients underwent preoperative thin-section chest CT scans and surgical resection. A total of 400 GGNs were included. Regions of interest (ROI) were delineated on the slice showing the largest diameter of the lesions. Based on pathological confirmation, the nodules were divided into two groups: Group 1 (adenocarcinoma in situ, AIS or minimally invasive adenocarcinoma, MIA, 209 nodules) and Group 2 (invasive adenocarcinoma, IAC, 191nodules). The dataset was randomly split into a training set (280 nodules, 70 %) and a validation set (120 nodules, 30 %) at a 7:3 ratio. In the training set, feature dimensionality reduction was performed using minimum redundancy maximum relevance (mRMR) as well as least absolute shrinkage and selection operator (LASSO) to screen out discriminative radiomics features. Then seven machine learning models—logistic regression (LR), support vector machine (SVM), random forest (RF), extra trees, XGBoost, GradientBoosting, and AdaBoost—were constructed. Model performance and prediction efficacy were evaluated based on indicators such as area under the curve (AUC), accuracy, specificity, and sensitivity using receiver operating characteristic (ROC) curves.

**Results:**

Eight radiomics features were ultimately identified. Among the seven models, the GradientBoosting model exhibited the best performance, achieving an AUC of 0.929 (95 % CI: 0.9004–0.9584), accuracy of 0.85, sensitivity of 0.851, and specificity of 0.849 in the training set.

**Conclusion:**

The GradientBoosting model based on CT radiomics features demonstrates superior performance in predicting pathological subtypes of ground glass nodular lung adenocarcinoma, providing a reliable auxiliary tool for clinical diagnosis.

Lung cancer is the malignant tumor with the highest incidence and mortality rate in China. According to pathological types, it is primarily classified into two major categories: non-small cell lung cancer (NSCLC) and small cell lung cancer (SCLC), with NSCLC accounting for over 80 % of cases[1]. Lung adenocarcinoma (LAC) is the predominant histological subtype of NSCLC[2]. According to the World Health Organization (WHO) pathological classification criteria, LAC is subdivided into three subtypes based on invasiveness: adenocarcinoma in situ (AIS), minimally invasive adenocarcinoma (MIA), and invasive adenocarcinoma (IAC). These subtypes exhibit distinct biological characteristics and prognoses[3], [4], [5]. As pre-invasive lesions, follow-up observation is the primary management approach for AIS. Both AIS and MIA demonstrate favorable prognoses after sublobar resection, whereas IAC is associated with a significantly higher postoperative recurrence rate[6], [7], Therefore, accurate identification and differentiation of the invasiveness levels in LAC are crucial for personalized treatment strategies and improving patient prognosis.

In recent years, the widespread application of low-dose computed tomography (LDCT) screening has significantly increased the detection rate of pulmonary nodules[8], Among these, malignant nodules account for less than 5 %. Based on the presence of solid components, pulmonary nodules are classified into Pure ground-glass nodules (pGGN), Mixed ground-glass nodules (mGGN), and pure solid nodules. pGGNs completely invisible on mediastinal window imaging, mGGNs partially visible, while pure solid nodules fully visible on mediastinal window[9]. Malignant nodules with ground-glass opacity features are predominantly pathologically diagnosed as adenocarcinomas. A retrospective study on GGNs demonstrated that [10], the maximum CT value measured on thin-section CT can help differentiate among AIS, MIA, and IAC. However, accurate identification of GGNs largely depends on radiologists' professional expertise and diagnostic proficiency[11], [12]. The definitive pathological classification can only be confirmed through invasive procedures such as surgical resection or needle biopsy[12], Therefore, developing a non-invasive approach based on preoperative CT imaging for early diagnosis and invasiveness assessment of GGNs holds significant clinical importance.

Machine learning (ML), a critical branch of artificial intelligence, demonstrates promising potential in the medical field by "learning" and "training" on large-scale sample datasets [13], [14], Commonly used ML algorithms include decision tree (DT), logistic regression (LR), random forest (RF), extreme gradient boosting (XGBoost), support vector machine (SVM), k-nearest neighbor (KNN) and etc.[15], [16]. Radiomics enables the extraction of high-throughput features from medical images to objectively quantify tumor heterogeneity. By integrating clinical characteristics, it facilitates tumor differential diagnosis, pathological classification, treatment response evaluation, and prognosis assessment [17]. The integration of radiomics with ML algorithms has also opened new avenues in oncology management.

Therefore, this study employs radiomics technology to extract quantitative features from CT images of LAC patients, constructing a machine learning model for early and accurate prediction of GGN invasiveness levels.

## 1 Materials and methods

### 1.1 Case selection and general characteristics

**Fig. 1:**
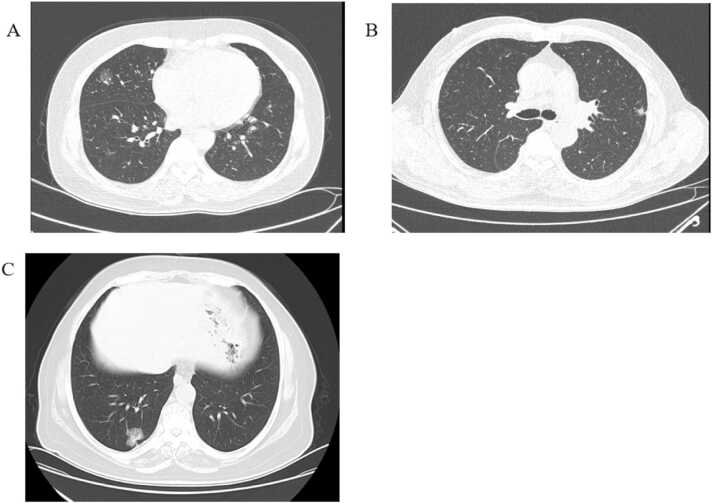
The CT images of all ground glass subtypes. A. The CT images of AIS; B. The CT images of MIA; C. The CT images of IAC.

This study retrospectively analyzed 392 LAC patients who underwent preoperative thin-section non-contrast chest CT followed by surgical resection at Binzhou Medical University Hospital between January 1, 2020, and May 31, 2023. A total of 400 GGNs were included, with 156 male and 236 female patients, aged 22–78 years. Inclusion criteria:(1) GGNs with ground-glass opacity (GGO) components on CT imaging. (2) Complete and diagnostically adequate images. (3) Postoperative pathological confirmation of LAC, including AIS, MIA, and IAC. (4) Comprehensive clinical records. Exclusion criteria: (1) Patients who received neoadjuvant radiotherapy or chemotherapy before surgery. (2) Patients who underwent needle biopsy prior to CT scanning. (3) Poor image quality affecting lesion delineation. (4) Images not meeting diagnostic requirements. (5) Incomplete clinical data or pathological results (Fig. 1).

### 1.2 Group classification

In this study, GGNs pathologically confirmed as AIS or MIA were classified as Group 1, while those diagnosed as IAC were designated as Group 2. The nodules in both groups were randomly divided into training and validation sets at a 7:3 ratio.

### 1.3 CT scanning protocol

All chest CT scans were performed using LightSpeed VCT, Brilliance CT, or Optima CT660 scanners. The scanning protocol was standardized as follows, patients were positioned supine with arms raised above the head and neck, adopting a head-first orientation. All scans were acquired at end-inspiration breath-hold. The scanning range extended from the lung apex to the base. Images were reconstructed using lung window settings (window width: 1500 HU; window level: −600 HU) for optimal parenchymal visualization [18], [19], The scanning parameters are detailed in Table 1.

**Table 1:** Scan parameters of different CT scanner models.

Scan parameters	Light Speed VCT	Brillinance CT	Optima CT660
Tube voltage (KV)	120	120	120
Tube current (mA)	180	180	180
Pitch	0.984	1.172	0.984
Collimator Width（mm）	40	40	40
Rotation time (s/r)	0.7	0.75	0.7
Field of view (cm^2^)	50 × 50	50 × 50	50 × 50
Reconstructed thickness (mm)	0.625	1	0.625
Reconstruction Interval (mm)	0.625	1	0.625
Reconstruction Algorithm	Lung reconstruction	Lung reconstruction	Lung reconstruction

### 1.4 Region of interest (ROI) segmentation and radiomic feature extraction

All CT images were exported via the PACS system in DICOM format and imported into the open-source medical imaging software 3D Slicer (version 4.10.2). ROIs (regions of interest) were manually delineated by a board-certified chest radiologist with 6 years of experience on thin-section lung window images (window width: 1500 HU; window level: −700 HU). The segmentation was performed on the axial slice showing the maximum lesion diameter, covering the entire tumor volume[20], Normal lung tissue, large blood vessels, and airways external to the GGN were excluded from the ROI. To ensure segmentation accuracy, all ROIs were independently reviewed and modified by a senior chest radiologist with 12 years of experience.

To minimize the impact of inter-image variability on the results, image normalization and resampling (resampling resolution: 1 mm × 1 mm × 1 mm) were performed prior to radiomics feature extraction[21]. Following image preprocessing, radiomic features were extracted from the lesions.

Eighty nodules were randomly selected, and two radiologists (with 6 years and 12 years of experience in thoracic imaging diagnosis, respectively) independently performd ROI delineation and extracted imaging features. The inter-class correlation coefficient (ICC) was used to assess the consistency of extracted imaging features, where an ICC ≥ 0.75 indicated good consistency[22]. The remaining lesions were delineated by intermediate-level radiologists and subsequently reviewed by senior radiologists for final confirmation.

### 1.5 Pathological evaluation

All pathological assessments were performed by pathologists with over 15 years of experience, strictly adhering to the 2021 World Health Organization (WHO) Classification of Thoracic Tumors diagnostic criteria[23].

### 1.6 Model development and validation

The optimal radiomic features were selected through a two-stage dimensionality reduction process implemented in Python. Firstly, performed feature dimensionality reduction using least absolute shrinkage and selection operator (LASSO), Further reduced feature redundancy via minimum redundancy maximum relevance (mRMR) algorithm and identified the most discriminative radiomic signature [24], [25]. Using the selected discriminative features as input variables, we constructed seven machine learning prediction models in the training set, including logistic regression (LR), support vector machine (SVM), random forest (RF), Extra Trees, extreme gradient boosting (XGBoost), Gradient Boosting, and Adaptive Boosting (AdaBoost)，All models were rigorously evaluated in the independent validation set using standardized performance metrics[26], [27].

### 1.7 Statistical method

**Fig. 2:**
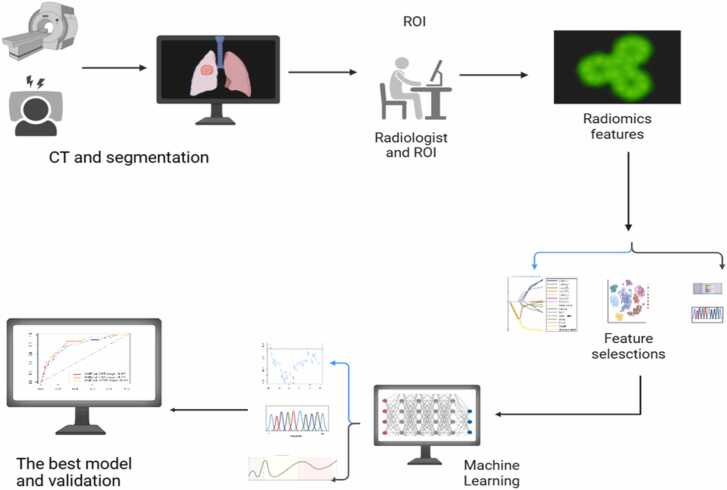
Technology Roadmap.

Statistical analyses were conducted using Python. Seven machine learning algorithms—LR, SVM, RF, XGBoost, Gradient Boosting, and AdaBoost—were employed to construct predictive models. The Performance Metrics of each model was evaluated using Area under the curve (AUC), sensitivity, specificity, and accuracy. Statistical significance was defined as a *p < 0.05*(two-tailed). Inter-observer consistency was assessed via ICC analysis, with an ICC ≥ 0.75 indicating good consistency. Overall study flowchart is shown in Fig. 2.

## 2 Results

### 2.1 General information

This study included 392 surgically resected LAC patients with a total of 400 GGNs, These nodules were stratified into Group 1 (n = 209) and Group 2 (n = 191), with maximum lesion diameters ranging from 6 to 36 mm. The dataset was randomly allocated into a training set (n = 280) and a validation set (n = 120) at a 7:3 ratio.

### 2.2 Reproducibility and consistency assessment

**Fig. 3:**
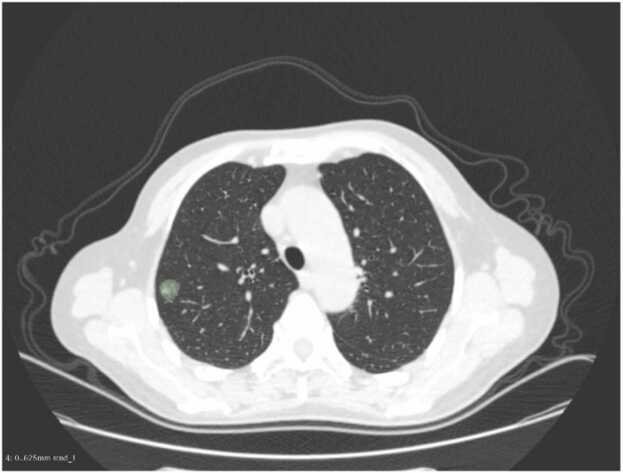
Delineation of region of interest.

Reproducibility analysis was performed on radiomic features extracted from ROIs of 80 GGN lesions by an intermediate-level radiologist (6 years of experience). A senior radiologist (12 years of experience) independently re-delineated the ROIs for the same 80 GGNs (Fig. 3). Compared all radiomic features using ICC, with ICC ≥ 0.75 defined as acceptable consistence.

### 2.3 The results of radiomic feature selection and model development

**Fig. 4:**
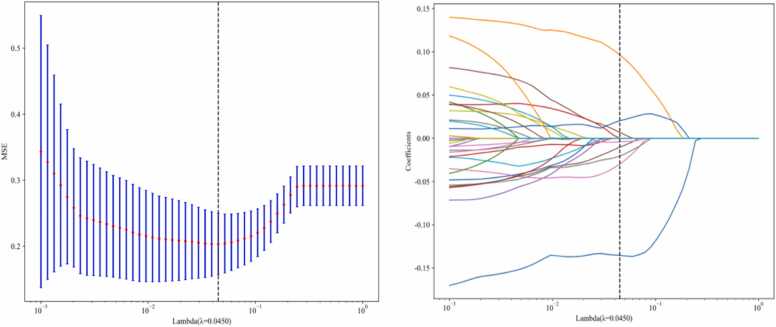
Radiomics Feature Screening Using LASSO Model. A. The feature set in the LASSO model, where the vertical dashed lines indicate the optimal lambda value (λ) selected through 10-fold cross-validation. B. The coefficient convergence plot of radiomic features in the LASSO model, where each curve represents the change trajectory of an individual predictor's coefficient.

**Fig. 5:**
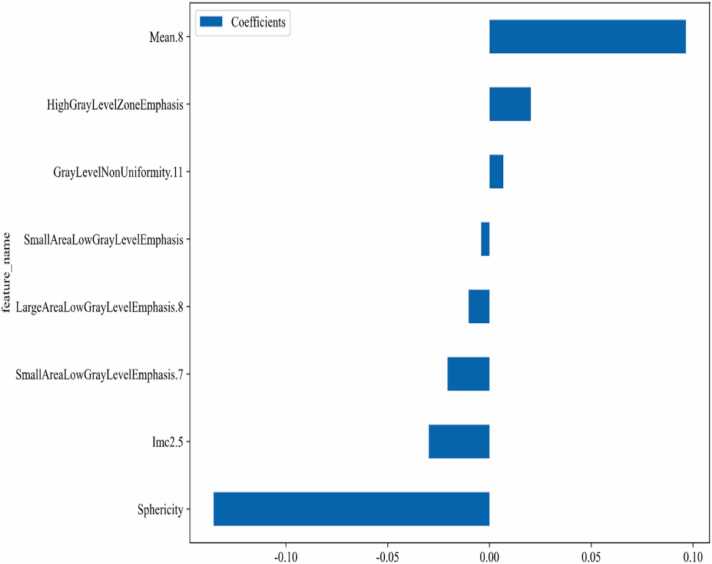
8 Radiomics Features Selected and their Corresponding Weight Values.

A total of 851 radiomic features were extracted, comprising 14 shape features, 162 first-order histogram features, and 675 texture features, Following dimensionality reduction via LASSO regression with 10-fold cross-validation and further redundancy reduction through the mRMR algorithm, 8 robust radiomic features were selected: Mean.8; High Gray Level Zone Emphasis; Gray Level Uniformity.11; Small Areal Low Gray Level Emphasis; Large Area Low Gray Level Emphasis.8; Small Areal Low Gray Level Emphasis.7; Imc2.5; Sphericity, The eight identified discriminative radiomic features were used to construct the predictive model (Fig. 4A and B), with the weight values of each feature shown in Fig. 5.

### 2.4 Construction of machine learning models

**Fig. 6:**
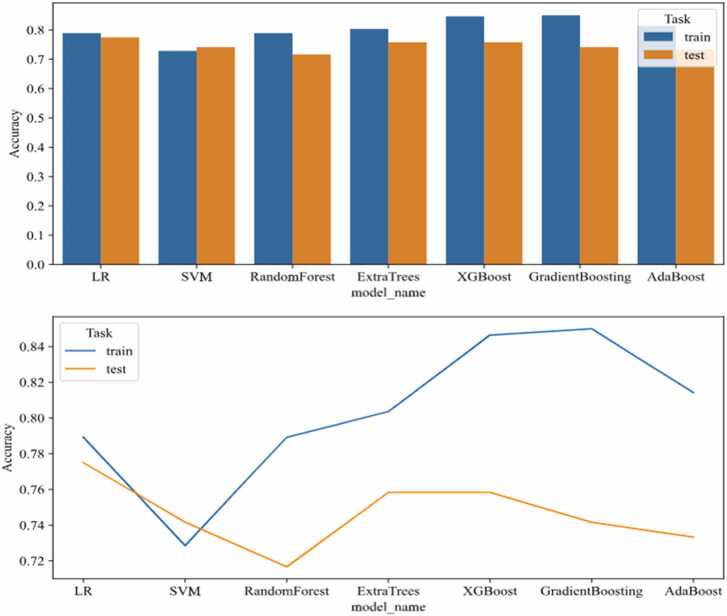
Accuracy comparison among different machine learning models.

Using the Scikit-learn package in Python, we constructed based on the optimal radiomic feature set in the training cohort: LR, SVM, RF, Extra trees, XGBoost, GradientBoosting, AdaBoost, Classification was performed using the refined radiomic features (Table 2). Among the seven models, Gradient Boosting demonstrated superior performance, achieving the highest AUC of 0.929 and the best accuracy of 0.85 in the training set (Fig. 6). Comprehensive evaluation confirmed Gradient Boosting as the optimal classifier.

**Table 2:** Accuracy, AUC, Sensitivity, Specificity, PPV, and NPV of Seven ML Models.

Group	ML Models	Accuracy rate	AUC	95 % CI	Sensitivity	Specificity	Positive predictive value (PPV)	Negative predictive value (NPV)
Training Set	LR	0.789	0.863	0.8201–0.9063	0.784	0.795	0.778	0.800
Validation Set	LR	0.775	0.842	0.7730–0.9118	0.684	0.857	0.812	0.750
Training Set	SVM	0.729	0.783	0.7300–0.8369	0.530	0.911	0.845	0.679
Validation Set	SVM	0.742	0.817	0.7398–0.8937	0.789	0.698	0.703	0.786
Training Set	RF	0.789	0.864	0.8227–0.9062	0.709	0.863	0.826	0.764
Validation Set	RF	0.717	0.800	0.7215–0.8781	0.702	0.73	0.702	0.730
Training Set	ExtraTrees	0.804	0.900	0.8658–0.9340	0.754	0.849	0.821	0.790
Validation Set	ExtraTrees	0.758	0.839	0.7697–0.9089	0.667	0.841	0.792	0.736
Training Set	XGBoost	0.846	0.924	0.8936–0.9538	0.858	0.836	0.827	0.865
Validation Set	XGBoost	0.758	0.830	0.7585–0.9023	0.842	0.683	0.706	0.827
Training Set	GradientBoosting	0.850	0.929	0.9004–0.9584	0.851	0.849	0.838	0.861
Validation Set	GradientBoosting	0.742	0.831	0.7592–0.9028	0.895	0.603	0.671	0.864
Training Set	AdaBoost	0.814	0.900	0.8664–0.9341	0.761	0.863	0.836	0.797
Validation Set	AdaBoost	0.733	0.791	0.7110–0.8716	0.772	0.698	0.698	0.772

### 2.5 Models validation

**Fig. 7:**
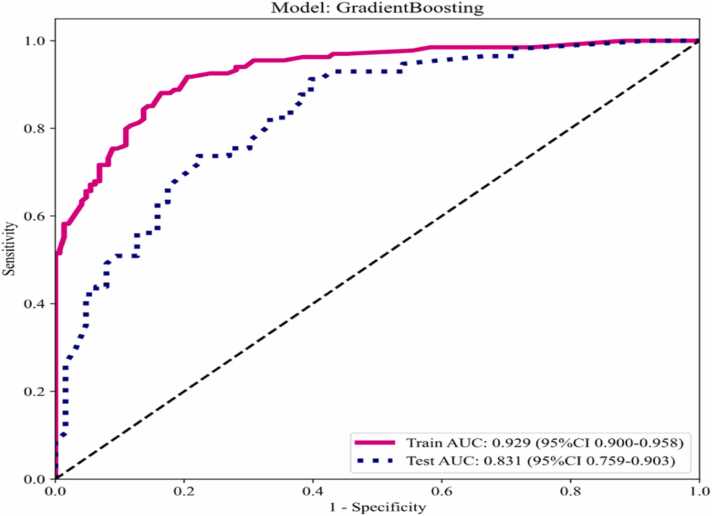
ROC Curve for Gradient Boosting Model Predicting Pathological subtyping of GGN.

The diagnostic performance evaluation of the optimal Gradient Boosting model was analyzed by ROC curve. The training set and the validation set were classified by 5-fold cross-validation, and the ROC curves of the Gradient Boosting model for predicting the training set and the validation set were obtained respectively (Fig. 7). The AUC values were 0.929 and 0.831 respectively, the accuracy rates were 0.85 and 0.742 respectively, and the sensitivities were 0.851 and 0.895 respectively. The specificities were 0.849 and 0.603 respectively, the PPV were 0.838 and 0.671 respectively, and the NPV were 0.861 and 0.864 respectively.

## 3 Discussion

The pathological classification and invasive potential of GGNs play crucial roles in formulating clinical management strategies, selecting appropriate therapeutic approaches and predicting long-term patient outcomes. Accurate histopathological classification serves as the foundation for developing effective treatment plans. Currently, the definitive diagnostic standard still requires invasive histopathological examination[28], [29]. Radiomics, as a powerful quantitative approach to characterize tumor features, bridges imaging features with clinical decision-making[30]. Consequently, developing a CT-based methods for predicting GGN histological classification holds substantial clinical value. Growing research demonstrates that radiomics and machine learning—as innovative approaches—outperform radiologist assessments in diagnostic accuracy, therapeutic decision support and longitudinal monitoring, which shows significant promise in revolutionizing pulmonary nodule management[31].

Chae et al. [32] utilized textural features to develop an artificial neural network for distinguishing non-invasive pulmonary nodules from invasive nodules, achieving an AUC of 0.98. However, this model may be limited by overfitting and questionable generalizability due to its small sample size (n = 86) and lack of external validation. Lu et al.[33] developed a model for differentiating invasive from indolent nodules achieved an AUC of 0.846, suggesting room for further improvement in diagnostic performance. Compared with previous studies, our research implemented several key advancements, selection of 8 optimal radiomic features from a larger training cohort (280 GGNs), application of improved feature selection methodology, and successful discrimination between AIS/MIA and IAC subtypes. These refinements significantly enhanced both the model's robustness and clinical applicability.

Among the eight selected radiomic features, Mean.8 (gray-level mean) and Sphericity demonstrated the highest contributions, as different nodules exhibited differences in gray-level distribution and irregularity on CT images. The former reflects the mean gray value of a specific region, which is used to assess intra-tumoral heterogeneity[34]. The latter quantifies the similarity of the three-dimensional shape to an ideal sphere, where higher sphericity suggests regular growth patterns potentially associated with benign lesions, whereas lower sphericity may correlate with invasive growth or malignant tumors [34]. A previous multi-dataset study identified that gray-level mean features are critical for distinguishing lung adenocarcinoma from squamous cell carcinoma [35], Additionally, research has shown that tumor sphericity serves as an independent predictor of overall survival in lung cancer patients undergoing standard treatment [36].

However, existing studies on GGN risk stratification demonstrate significant heterogeneity in selected radiomic features and model performance, which may be attributed to methodological differences and dataset heterogeneity. Previous studies have developed diverse models to predict cellular differentiation and invasiveness in GGNs. Due to variations in methodologies and the complexity of multifactorial clinical data, the importance and predictive power of these variables in models also exhibit significant discrepancies. Balagurunathan et al. achieved an AUC of 0.83 for predicting malignant nodules by analyzing radiomic texture features of non-morphological characteristics in pulmonary nodules [37]. Similarly, Mao et al. constructed a classifier using 11 radiomic features to predict solid pulmonary nodules, reporting a sensitivity of 0.922 and specificity of 0.810 [38]. Although both studies employed the same logistic regression approach, their model performances differed markedly.

To enhance the accuracy of GGN risk prediction and identify the optimal predictive model, we considered the limitations of conventional logistic regression in addressing collinearity among independent variables, while the LASSO regression approach effectively mitigates inter-variable multicollinearity [39]. Consequently, in this study, we employed LASSO regression to identify 8 key radiomic features, subsequently constructing and comparing predictive models using 7 distinct ML algorithms for comparative analysis. All models were evaluated using metrics including accuracy, sensitivity, specificity, PPV, NPV, and ROC analysis.

The results demonstrated that the Gradient Boosting model outperformed others in the training set, achieving superior metrics (accuracy: 0.85, sensitivity: 0.851, specificity: 0.849, PPV: 0.838, NPV: 0.861). This model also demonstrated superior in AUC values in both the training set (AUC = 0.929) and validation set (AUC = 0.831).

LR, SVM, RF, Extra trees, XGBoost, AdaBoost are prone to overfitting, where they perform well on training data but fail to generalize to unseen data, leading to inflated performance metrics Additionally, the lack of interpretability of these models, which operate as “black boxes”, can hinder trust and acceptance among healthcare professionals, limiting their clinical utility. Gradient Boosting can boost a weak learning algorithm with an accuracy slightly better than random guessing into an arbitrarily accurate strong learning algorithm, bringing about a new method and new insights into the design of the learning algorithm. Even if many base classifier instances are used, Gradient Boosting rarely overfits the solution and minimizes the exponential loss function by fitting the stepwise additive model.

Although Gradient Boosting model’s performance slightly declined in the validation set, this may be attributed to the limited sample size included in the study. Therefore, we conclude that the Gradient Boosting model exhibits exceptional overall performance in accurately predicting the histological subtypes of GGNs.

The biggest advantage of radiomics is that these high-dimensional quantitative features can be combined with machine learning to build predictive models. In the construction of radiomics-based prediction models, the selection of suitable machine learning algorithms can enhance the predictive accuracy and stability of the model. Recent advancements in machine learning algorithms, including Gaussian processes, Extra Trees, RF, SVM, GradientBoosting, and LR, have propelled the application and development of radiomics. Shen [40] evaluated seven different classifiers to optimize a model for classification. The study found that a PET/CT radiomics model using the SVM classifier demonstrated the best performance in NSCLC, with an AUC of 0.9155. Parmar et al. [41] demonstrated that the RF method was the most effective in managing radiomic feature instability, outperforming 12 other machine learning classifiers, including XGBoost, AdaBoost and SVMs—in terms of prognostic performance. Huang et al. [42] employed the GBM algorithm to develop both a radiomic model and a fusion model (clinical + radiomic) to predict EGFR mutation status in patients with NSCLC.

Machine learning can be broadly categorized into two main types: supervised learning and unsupervised learning. Supervised learning generally yields better learning outcomes and is widely employed in radiomics analysis. Therefore, this study selected common supervised algorithms and compared their diagnostic performance, aiming to identify the optimal model. Due to the different algorithms and working principles of each single classifier, the sensitivity of different classifiers to different data sets is different, which leads to differences in their predictive performance. LR is a classification model based on linear regression. Its advantages lie in computational simplicity and strong interpretability; however, it performs poorly on some non-linear data[43]. The SVM algorithm classifies features by searching for the optimal hyperplane that separates different classes in the feature space. It is less dependent on data dimensionality and sample size[44]. DT (Decision Tree) classifies data through "yes" or "no" answers. It is only suitable for small-scale datasets. RF is an ensemble algorithm based on DT, offering better accuracy than individual algorithms, fast data processing speed, the capability to handle large-scale data without computational penalties, and is naturally resistant to overfitting[45]. Extra Trees, a variant of the RF, enhances model generalization by introducing greater randomness to reduce variance. It can also accelerate training speed to some extent. However, the increased randomness may lead to potential instability in model performance[46]. XGBoost's core mechanism integrates multiple weak classifiers (primarily decision trees) into a strong predictive model through gradient boosting strategy. It consistently demonstrates outstanding performance in both classification and regression tasks [47]. AdaBoost sequentially constructs a series of weak learners and combines them into a strong learner. Its core mechanism lies in adjusting sample weights such that each new model specifically targets samples misclassified by preceding models, thereby continuously improving overall model performance [48]. Gradient Boosting incrementally builds a series of weak learners by optimizing the model's loss function through gradient descent. This yields a powerful ensemble model. The core mechanism lies in leveraging gradient information to enhance predictive performance, continuously minimizing residuals to improve the overall model [49].

This study incorporated a broader representation of pulmonary nodule types, which not only enhances the extraction of radiomic features but also better aligns with clinical applicability. Furthermore, prior to radiomic feature extraction, we normolized and resampled CT images to mitigate the impact of variability in scanning equipment, acquisition parameters, or reconstruction algorithms on the reproducibility of radiomic features. Through rigorous reproducibility testing, only features satisfying both intra- and inter-observer ICC≥ 0.75 were retained. This approach further ensures feature stability and strengthens the robustness and reliability of the study findings.

While this study validates the potential of radiomic models in predicting the pathological subtypes of GGNs, several limitations remain: (1) Limited Sample Scope: The relatively small sample size, derived from a single-center retrospective cohort, exclusively included surgically resected lung adenocarcinoma patients. The exclusion of benign lesions or other malignant tumor types may introduce selection bias. (2) Manual Segmentation Variability: Although ROIs were manually delineated and reviewed by experienced radiologists, the variability in segmentation was not quantitatively assessed. Future work should prioritize developing automated segmentation algorithms to minimize human error and improve reproducibility. (3) Incomplete Feature Integration: The exclusion of contrast-enhanced CT imaging features may overlook the potential value of hemodynamic information (e.g. perfusion characteristics) in assessing tumor invasiveness. (4) Perinodular Feature Exploration: The current model does not incorporate perinodular radiomic features (e.g., peri-tumoral microenvironmental textures), which may provide critical insights into comprehensive invasiveness prediction.

In conclusion, radiomics-based ML models utilizing CT features offer a non-invasive and efficient tool for the pathological classification of GGNs. The Gradient Boosting model developed in this study demonstrated superior performance in distinguishing AIS/MIA from IAC, serving as a valuable complement to invasive pathological examinations. Despite challenges such as sample bias and segmentation standardization, this model provides critical insights for formulating preoperative individualized treatment strategies, thereby advancing the clinical implementation of precision medicine in GGN management.

## CRediT authorship contribution statement

**Rui Chen:** Writing – review & editing, Resources, Project administration, Methodology, Formal analysis, Data curation. **Hu Zhang:** Software, Resources, Methodology, Investigation, Formal analysis, Data curation. **Xingwen Huang:** Writing – review & editing, Validation, Supervision, Project administration. **Haitao Han:** Writing – review & editing, Supervision, Investigation, Data curation, Conceptualization. **Jinbo Jian:** Writing – review & editing, Validation, Supervision, Funding acquisition.

## Ethical statement

The protocol was approved by Ethics Committee of Binzhou Medical University Hospital.

## Funding

This study was supported by the Shandong Medical and Health Science and Technology Development Project (No. 202009010691).

## Declaration of Competing Interest

The authors declare that the research was conducted in the absence of any commercial or financial relationships that could be construed as a potential conflict of interest.
